# Precision Health Economics and Outcomes Research to Support Precision Medicine: Big Data Meets Patient Heterogeneity on the Road to Value

**DOI:** 10.3390/jpm6040020

**Published:** 2016-11-02

**Authors:** Yixi Chen, Gregory F. Guzauskas, Chengming Gu, Bruce C. M. Wang, Wesley E. Furnback, Guotong Xie, Peng Dong, Louis P. Garrison

**Affiliations:** 1 Medical, Pfizer Investment Co. Ltd., Beijing 100010, China; YiXi.Chen@pfizer.com (Y.C.); ChengMing.Gu@pfizer.com (C.G.); Peng.Dong@pfizer.com (P.D.); 2 Department of Pharmacy, University of Washington, Seattle, WA 98195, USA; greguz@uw.edu (G.F.G.); lgarrisn@uw.edu (L.P.G.); 3 Elysia Group, LLC, New York, NY 10016, USA; Wes.furnback@elysiagroup.com; 4 IBM Research, Beijing 100010, China; XIEGUOT@cn.ibm.com

**Keywords:** big data, precision health economics, precision medicine

## Abstract

The “big data” era represents an exciting opportunity to utilize powerful new sources of information to reduce clinical and health economic uncertainty on an individual patient level. In turn, health economic outcomes research (HEOR) practices will need to evolve to accommodate individual patient–level HEOR analyses. We propose the concept of “precision HEOR”, which utilizes a combination of costs and outcomes derived from big data to inform healthcare decision-making that is tailored to highly specific patient clusters or individuals. To explore this concept, we discuss the current and future roles of HEOR in health sector decision-making, big data and predictive analytics, and several key HEOR contexts in which big data and predictive analytics might transform traditional HEOR into precision HEOR. The guidance document addresses issues related to the transition from traditional to precision HEOR practices, the evaluation of patient similarity analysis and its appropriateness for precision HEOR analysis, and future challenges to precision HEOR adoption. Precision HEOR should make precision medicine more realizable by aiding and adapting healthcare resource allocation. The combined hopes for precision medicine and precision HEOR are that individual patients receive the best possible medical care while overall healthcare costs remain manageable or become more cost-efficient.

## 1. Introduction

Personalized medicine has historically been associated with human and disease genetics, which have unlocked the ability to target medicines to the patients most likely to benefit. The terms “precision” or “stratified” medicine have emerged in recent years as a refinement of this concept [1,2]. This newer concept was formally defined by President Obama’s 2015 Precision Medicine Initiative as “an approach to disease treatment and prevention that seeks to maximize effectiveness by taking into account individual variability in genes, environment, and lifestyle” [3]. More recently, Jameson and Longo defined precision medicine as “reatments targeted to the needs of individual patients on the basis of genetic, biomarker, phenotypic, or psychosocial characteristics that distinguish a given patient from other patients with similar clinical presentations” [4].

These expanded definitions mention non-genetic characteristics and terms such as “lifestyle”, “psychosocial”, and “effectiveness”. However, neither definition explicitly mentions costs, cost-effectiveness, or value. What, then, is the relationship of health economics and outcomes research (HEOR) to precision medicine? Furthermore, there is an important third factor—big data—that is altering this entire development and translation process.

Big data lacks a universal definition, but has been previously defined by several publications [5,6,7]. Krumholz has perceptively argued: “Big data in medicine—massive quantities of healthcare data accumulating from patients and populations and the advanced analytics that can give those data meaning—hold the prospect of becoming an engine for the knowledge generation that is necessary to address the extensive unmet information needs of patients, clinicians, administrators, researchers, and health policy makers” [8]. Several examples of big data sets in healthcare are described by Raghupathi and Raghupathi [9].

We agree entirely with this sentiment, and believe that the idea of “precision HEOR” is worth exploring as an important link in the translation of big data–driven precision medicine into clinical practice through improved health sector decision-making. In the spirit of offering formal definitions, we propose that precision HEOR utilizes a combination of costs and outcomes derived from big data to inform healthcare decision-making that is tailored to highly specific patient clusters or individuals. In this commentary, we will attempt to elucidate the potential relationships among HEOR, precision medicine, big data, and improved health system value.

## 2. The Role of HEOR in Health Sector Decision-Making

**Figure 1:**
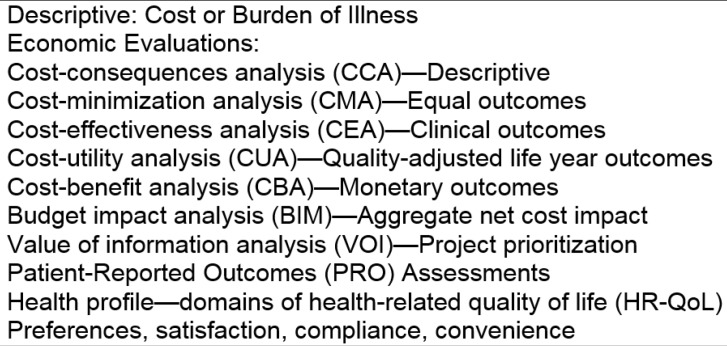
Types of health economics and outcomes research (HEOR) studies.

HEOR attempts to assess the “value” of health system changes and interventions by comparing their outcomes with their costs. The overall rationale is that given limited resources to devote to health, we want to maximize their beneficial impacts on the population. Traditional HEOR has focused on projecting either the likely cost-effectiveness or likely budget impact when a new medicine or other intervention is adopted and launched in the healthcare system. The HEOR catalogue has expanded in recent years to include other analyses such as burden of illness and value of information studies, among others (Figure 1). Model-based analyses utilize the best available evidence from representative populations, and assess the likely economic value of a treatment in the context of the typical patients in the study population. The utilized evidence base includes clinical evidence (i.e., efficacy, safety, quality), economic factors (i.e., cost, coverage, and reimbursement), and patient-centered outcomes (i.e., patient preferences and quality of life).

For the purposes of this discussion, HEOR can be separated into its outcomes research (OR) part, which is concerned with measuring clinical impact, impact on patient quality of life, and ultimately on both quality and length of life (i.e., quality-adjusted life years (QALYs)), and its health economics (HE) part, which is concerned with measuring incremental cost impact. The cost-effectiveness (or value) of an intervention combines the OR and HE in the incremental cost-effectiveness ratio (ICER), which compares the difference in cost versus the difference in effectiveness between two (or more) competing interventions. The ICER can be compared to a societal “willingness to pay” threshold to determine whether or not an intervention should be provided to particular types of patients.

Although traditional HEOR uses elements of what some would consider to be big data (e.g., large administrative claim data and epidemiological data), it has two major limitations with respect to the broader potential for big data that many envision in precision medicine. First, although traditional HEOR sometimes uses administrative claims or observational data sets to supplement clinical trial data, the ability of the analysis to predict outcomes or costs precisely for specific types of individuals is limited by the sample size of patients with clinical trial data. The second limitation is that HEOR models aim to project effectiveness (in the real world) based on a mixture of efficacy data (from trials) supplemented with some external data from observational databases. At best, traditional HEOR models are an early projection of likely effectiveness in the real world. It should be obvious that this is very different from the amount of information that might become available, for example, after 100,000 patients have received a particular intervention and their experience is captured in electronic health records, which can influence decision-making.

## 3. Big Data and Predictive Analytics

Big data in healthcare refers to huge health-related data sets and their associated predictive analytics [10,11]. Common uses for big data include: providing population characteristics; identifying risk factors and developing prediction (diagnostic or prognostic) models; observational studies comparing different interventions; exploring variation among healthcare providers; and as a supplementary source of data for another study [12]. The main advantages of big data analyses are their comprehensive nature, the immense populations they can accommodate, and the ability to compare healthcare providers. The main challenges are demonstrating data quality, the difficulty in applying a causal interpretation to the study findings, and a non-willingness by stakeholders such as healthcare providers to accept new methods [12,13].

Currently, big data is most closely associated with electronic medical record databases and claims-based databases [14]. Widespread transitions to electronic medical records have generated massive data sets of quantitative data such as laboratory values, qualitative data such as demographics, and expenditure data. However, much of the data is currently perceived as a by-product of healthcare delivery, rather than a central asset to improve its efficiency [15]. Some believe that by leveraging the collection of patient and practitioner data, health systems can use this data-driven approach to improve the quality and efficiency of healthcare delivery. As the number of electronic medical records increases, the potential for making better resource allocation decisions is expected to increase [16]. There is also a need for a consistent and efficient linkage between claims and outcomes data from a variety of settings. This linkage will need to aggregate data from patients, physicians, payers, and other stakeholders in patient care.

The big data sources that can be helpful in health sector decision-making go beyond traditional electronic health records, larger and longer claims databases, and surveys. The Internet combined with powerful machine learning analytics open up a variety of new avenues for enriching patient and provider data [17]. First, devices that produce continuous real-time read-outs of biomarkers, such as glucose levels, generate massive amounts of individual-level data. Second, choices made by patients searching the Internet can give clues to emerging diseases, such as influenza. Third, the choices that patients make as healthcare consumers can provide data for predictive analytics to predict their other health behaviors. Fourth, providers can be enlisted, e.g., in disease registries, to enter supplemental data about prescriptions that can be used to track outcomes both for different types of patients and for specific providers. Finally, the genomics revolution supported by this computing power makes it possible to sequence individual genomes, generating massive amounts of new data for patients and populations.

One current big data methodology that can make HEOR more precise is a technique known as “patient similarity analysis”. Patient similarity analysis aims to identify patients who experience a particular health outcome of interest and display similar clinical characteristics, risk factors, and treatment pathways [18]. The algorithmic approach first collects data from a large sample of patients who experience the outcome. Similar patients are identified using a flexible baseline similarity metric framework that combines information from heterogeneous sources of information such as electronic medical records, demographics, and genetics. Based on the available patient information, the algorithm then groups patients into clusters with similarities that may go unnoticed by human assessment. Next, each patient cluster is phenotyped to decipher inter-cluster differences and risk factor heterogeneity. Finally, the algorithm performs decision rule mining to map the most optimal treatment pathways for future patients based on the type of patient and the clinical strategies that showed the greatest benefit for each cluster. The resulting individualized patient insight is a multipurpose in scope: it enables personalized treatment, provides future risk assessment, and limits the costs associated with suboptimal care.

## 4. Making HEOR More Precise in Different Health Sector Decision Contexts

Traditional HEOR for new medicines has focused on the typical patient and often in terms of the mean, median or mode. This focus fails to recognize the real-world heterogeneity in patient populations and can be inefficient when using “one-size-fits-all” solutions. Furthermore, for many health sector decisions, the mean (rather than median) response or prediction is the more relevant one for clinical or economic policymaking. This strategy often fails to identify patient subgroups that potentially could have significantly more benefit than the general patient population.

**Figure 2:**
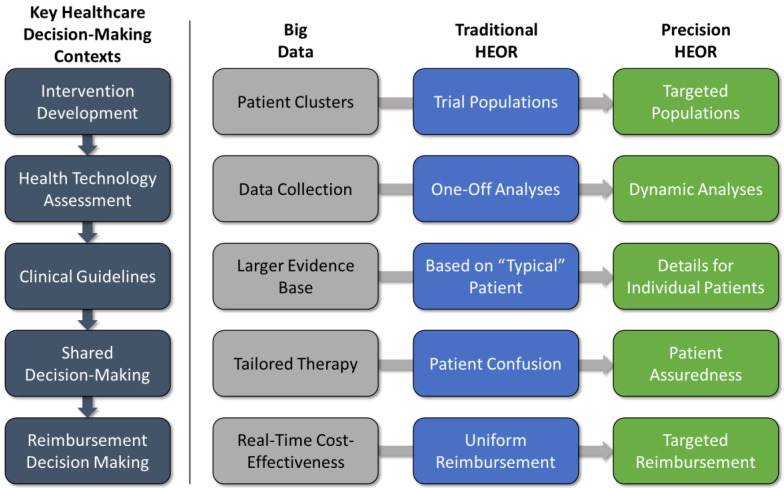
Big data impacts on HEOR.

There are several contexts in which traditional HEOR studies can play a role in key healthcare decision-making processes leading to real-world implementation: (1) development and testing of the intervention in clinical trials; (2) health technology assessment (HTA) decisions about coverage and reimbursement; (3) development of clinical practice guidelines; (4) patient-provider shared decision-making; and (5) reimbursement decision-making. Let us briefly consider how big data and predictive analytics might affect the application of HEOR within the key decision contexts (Figure 2).

### 4.1. Intervention Development

During a new intervention’s development, one of the main objectives for HEOR is to examine the potential value of a new intervention prior to product launch. These analyses either are performed alongside clinical trials or are done shortly thereafter, utilizing trial results, and are typically limited to modeling primary efficacy outcomes, as mentioned above. Augmenting this approach with more specific clinical characteristics, risk factors, treatment pathways, and genomic data can further define different patient cluster subgroups with differential responses, leading to a more nuanced value story.

### 4.2. Health Technology Assessment (HTA)

Decision model results are often considered in both price negotiations with payers and their decisions regarding formulary placement (i.e., with respect to inclusion and utilization management). As new products move into the real world, it is clearly important for everyone involved to learn how they work best and for which patients, and to make differentiated reimbursement decisions accordingly. Unfortunately, post-launch data collection is a costly procedure that has not fully taken hold in recent years. If the advent of big data analytics can reduce the cost of the data collection, models should transition away from “one-off” snapshots of an intervention’s value toward more dynamic analyses capable of adjusting to new patient clusters as they are identified. Of note, this practice may have strong implications for product life cycles as the availability of real-world data influences decision-making.

### 4.3. Clinical Guidelines

Clinical guidelines play an important role in health systems as they represent the current state of scientific knowledge, offering consensus judgments that give providers guidance as to the range of acceptable clinical pathways for a given disease condition. Clinical trials remain the gold standard for evidence in guideline development. However, for practical and cost reasons, these trials are typically focused on assessing efficacy, and are underpowered to measure adverse events. Guideline developers are ideally responsible for assessing both the safety and benefit-risk balance of a treatment in addition to efficacy. Guideline decision-making using precision HEOR would allow a full accounting of the benefits and risks of a new intervention. Furthermore, costs and resource utilization have not been explicitly considered in clinical guidelines [19]. Precision HEOR approaches could facilitate greater specificity in terms of both individual patient variability and value in future clinical guidelines.

### 4.4. Patient-Physician Shared Decision-Making

When the time comes to make a clinical decision, the physician as expert advisor must communicate with the patient both in terms of projecting likely clinical outcomes for alternative treatments, and in terms of understanding patient preferences for alternative outcomes. Rather than confusing the patient with multiple treatment options with varying success rates and costs, precision HEOR will present the patient with the most cost-effective treatment based on her/his defining characteristics, instilling confidence going forward in the treatment process.

### 4.5. Reimbursement Decision-Making

Traditional HEOR suggests the same reimbursement level for all patients with the same disease, whereas precision HEOR is able to support different reimbursement levels for targeted patient populations by utilizing more dynamic decision analysis techniques fed by big data analytics.

## 5. Moving Toward Precision HEOR

Big data applications to HEOR have the potential to transform healthcare delivery in positive and negative ways [12]. For healthcare payers, the transformation to optimized individual treatments can reduce costs by avoiding the sequelae foregoing the treatment odyssey of suboptimal treatments. Drug and medical device manufacturers will benefit from being able to identify relevant patient populations for niche market access strategies without the need for many expensive and time-consuming trials.

Initially at least, as the march to precision medicine gathers momentum, not much will change in how HEOR research is done, except that patients will be studied in specific clusters identified through patient similarity analysis. We assume that HEOR researchers will generally have more accurate models due to greater data access, fewer limiting assumptions, and greater data precision. Through machine learning, HEOR researchers will come to utilize real-world outcomes data on specific subpopulations and on clinical pathways that were unknown and/or had no prior clinical trial evidence. Importantly, these real-world data have high external validity compared to clinical trial data. In addition, the potentially huge population sizes utilized in big data analyses should greatly reduce the statistical uncertainty in model parameters, provided the biases of non-randomized research are overcome. A far-sighted approach to modeling that current HEOR researchers should begin to consider is the incorporation of dynamic model input methods that can incorporate streams of new patient data as it is made available (so-called “stream computing”).

Another benefit of precision HEOR will be the ability to better assess economic value for so-called orphan interventions, which often appear to offer low value compared to other treatments, but may be ideal for subgroups of patients. A traditional HEOR model estimates the favorability of a treatment versus competitors, and if the ICER is favorable and sensitivity analysis results are robust, an unequivocal recommendation is made to opt for the new treatment and discard the old one. However, trial evidence is rarely, if ever, 100% in favor of one treatment versus another, and economic analyses often indicate less than 100% cost-effectiveness acceptability under a given monetary threshold. The implication is that the suboptimal treatment (from a population standpoint) still has value for some patients.

However, big data and precision HEOR have some limitations. For one, the amount of data available for patient similarity analysis and the findings accessible to HEOR researchers will be massive, implying large costs for data storage. Another concern will be the inevitable questions regarding a comparative lack of scientific rigor compared to clinical trial evidence (and the potentially high probability of spurious findings) [12]. Third, several barriers to entry exist, and these include those from stakeholders, and general logistical concerns. Buy-in from clinicians, many of whom may be unwilling to relinquish or at least share clinical decision-making to a computer algorithm, and health plans will be crucial to the big data transition. Logistically, the linking of different databases between stakeholders in different settings such as physicians, patients, and payers, concerning different types of data (clinical and economic), must be configured. Fourth, there will always remain a difference between sample size and true “precision”; ultimately, every individual is unique and big data will never be able to capture all relevant patient characteristics in the model. Lastly, big data analysis might lead to the unwanted release of personal data unless proper safeguards are in place.

As precision HEOR matures as a field, all current healthcare stakeholders might face changes in their daily activities that collectively work towards a learning and more dynamically efficient healthcare system. Most explicitly, it is expected that big data–based disease risk prediction models will forecast multiple treatment options and outcomes, and then precision HEOR will evaluate high-precision economic impacts of disease prevention or early intervention in real time. That is, the optimization of the clinical decision supporting system will include automatic recommendations for the most cost-effective treatment to an individual patient based on his/her unique characteristics. This development will not only transform physicians’ work model to a data-driven paradigm, but is also likely to please patient advocates, as precision HEOR will facilitate patients’ joint decision-making with physicians about the most beneficial treatment for him/herself.

For healthcare payers, precision HEOR means improved reimbursement decision-making based on individual patient characteristics, and in different locations, which will be particularly useful for provincial or city-level payers. The utilization of precision HEOR decision-making will help payers avoid the costs associated with therapies predicted to have low success rates for individual patients. The net effect for a health system will be better healthcare resource allocation for greater clinical and economic value.

## 6. Conclusions

Fundamentally, precision medicine cannot exist without big data, and precision HEOR seems to be the natural complement to precision medicine. Even though big data makes the possibilities for optimizing treatment seem limitless, healthcare resources are not. Efforts to efficiently manage scarce health sector resources must continue in the big data era. Therefore, we conclude that precision HEOR will likely evolve to replace traditional HEOR given enough time. Precision HEOR can help to realize the promise of precision medicine by informing more efficient healthcare resource allocation. The combined hopes for precision medicine and precision HEOR are not only that individual patients receive the best possible medical care for their situation, but also that overall societal healthcare resource allocation will achieve the best outcomes for the money spent.
